# Outcomes of Intravenous Normal Saline Infusion Pre‐Cardiac Implantable Electronic Devices Versus No Infusion in Fasting

**DOI:** 10.1111/anec.70139

**Published:** 2025-12-15

**Authors:** Muhammad Hanzla Umair, Shahab Saidullah, Sadaf Shabeer, Roha Daneyal, Neha Kumar, Priyanka Shetiya, Hina Ahmed Siddiqi, Haresh Kumar, Anjali Bai, Raja Sadam Mehmood, Abida Perveen, F. N. U. Abdullah, Jahanzeb Malik

**Affiliations:** ^1^ Department of Medicine Ibn e Seena Hospital Kabul Afghanistan

**Keywords:** cardiac implantable electronic device (CIED), intravenous normal saline, procedural complications, venous puncture failure

## Abstract

**Background:**

Venous puncture failure during cardiac implantable electronic device (CIED) implantation is a significant procedural challenge, particularly in fasting patients. Pre‐procedural intravenous normal saline (NS) infusion may enhance venous filling and improve procedural outcomes, but evidence in this setting is limited.

**Methods:**

We conducted a retrospective cohort study at Abbas Institute of Medical Sciences, including 2852 patients undergoing CIED implantation. Patients were divided into two groups: those who received intravenous NS infusion prior to the procedure (*n* = 1130) and those who did not (*n* = 1722). Baseline demographics, procedural details, and outcomes—including venous puncture failure, arterial puncture, site change, and acute kidney injury (AKI)—were compared.

**Results:**

The NS group demonstrated a significantly lower rate of venous puncture failure (4.6% vs. 8.9%, *p* < 0.001) and arterial puncture failure (1.6% vs. 2.8%, *p* = 0.03). AKI occurred less frequently in the NS group, although this difference was not statistically significant (1.8% vs. 2.6%, *p* = 0.09). Predictors of venous puncture failure included absence of NS infusion (OR 2.1, 95% CI 1.5–3.0), BMI ≥ 30, and CKD. ROC analysis demonstrated good model discrimination (AUC = 0.81).

**Conclusion:**

Pre‐procedural NS infusion significantly improves venous puncture success in fasting patients undergoing CIED implantation.

## Graphical abstract

Pre‐procedural normal saline infusion significantly reduced venous puncture failure and arterial puncture in 2852 fasting CIED patients, with trends toward lower AKI. Absence of NS, high BMI, and CKD predicted failure, and model performance was strong (AUC 0.81). NS infusion appears to enhance procedural success.

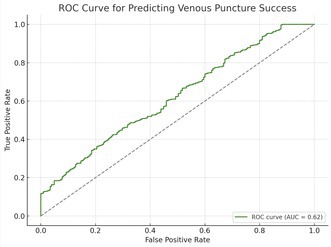

## 1 Introduction

Cardiac implantable electronic devices (CIEDs), such as pacemakers and implantable cardioverter‐defibrillators (ICDs), are cornerstone therapies in the management of bradyarrhythmias, conduction disorders, and prevention of sudden cardiac death (Steffen et al. 2019). These procedures require central venous access, typically via the subclavian, axillary, or cephalic vein, to allow placement of transvenous leads. Achieving successful venous puncture is a critical step in device implantation and can be technically challenging, especially in patients with low intravascular volume status (Atti et al. 2020).

Pre‐procedural fasting is routinely practiced to minimize the risk of aspiration in patients undergoing CIED implantation under local anesthesia with or without sedation. However, fasting may lead to intravascular volume depletion, venous collapse, and reduced venous distension, making venous access more difficult. This may result in increased venous puncture attempts, prolonged procedure times, accidental arterial puncture, or even procedural failure (Rüggeberg et al. 2024).

Intravenous fluid administration before vascular access is a well‐established strategy in various interventional settings to optimize venous filling and improve the success rate of cannulation (Ho et al. 2022). Normal saline (NS) infusion in fasting patients may help restore venous capacitance, increase venous diameter, and facilitate easier access. Despite its physiological plausibility, limited data are evaluating the role of pre‐procedural NS infusion in improving venous access outcomes in patients undergoing CIED implantation (Zhou et al. 2018).

Given the importance of first‐attempt venous puncture success in minimizing complications and enhancing procedural efficiency, identifying modifiable factors such as volume status becomes crucial. In particular, a simple intervention like pre‐procedural saline infusion could offer significant benefits in selected populations (Malbrain et al. 2020).

Therefore, the objective of this retrospective study is to compare venous puncture failure rates in fasting patients undergoing CIED implantation who received intravenous normal saline infusion versus those who did not.

## 2 Methods

### 2.1 Study Design and Setting

This retrospective observational study was conducted at Abbas Institute of Medical Sciences (AIMS), a tertiary care center with an active cardiac electrophysiology and device implantation program. The institutional review board (IRB) approved the study under study ID: AIMS/25/14. Experienced cardiac electrophysiologists performed all procedures in the cardiac catheterization laboratory.

### 2.2 Study Population

We included adult patients (age ≥ 18 years) who underwent cardiac implantable electronic device (CIED) implantation—either a pacemaker or an implantable cardioverter‐defibrillator (ICD)—between January 2020 and December 2024. Patients were included if they were fasting for at least 6 h before the procedure and had complete procedural documentation. Exclusion criteria included:

- Emergency procedures
- Pre‐existing central venous catheter or dialysis catheter
- Procedures using surgical venous cutdown
- Missing data on intravenous fluid administration or venous puncture attempts

### 2.3 Study Groups

Patients were divided into two groups based on pre‐procedural management:

- NS Group: Patients who received intravenous normal saline (500–1000 mL) within 2 h before the procedure.
- No‐NS Group: Patients who remained fasting and did not receive any pre‐procedural intravenous fluids.

The decision to administer IV fluids was at the discretion of the attending physician and anesthetist based on clinical judgment.

### 2.4 Outcomes

The primary outcome was *venous puncture failure*, defined as:

- More than two attempts at venous access, or
- Need to switch to an alternative access site (e.g., from subclavian to axillary vein or site change)

Secondary outcomes included:

- Procedure duration (skin‐to‐skin time)
- Incidence of arterial puncture
- Incidence of pneumothorax or hemothorax
- Intra‐procedural hypotension (SBP < 90 mmHg)
- Acute kidney injury (as per KDIGO criteria)

### 2.5 Data Collection

Patient records, procedural notes, and nursing charts were reviewed. Data collected included demographics, comorbidities (e.g., diabetes, CKD, heart failure), type of device implanted, volume and timing of fluid administration, number of venous puncture attempts, access site used, and procedural complications.

### 2.6 Statistical Analysis

Continuous variables were expressed as mean ± standard deviation or median (IQR) and compared using Student's *t*‐test or Mann–Whitney *U*‐test as appropriate. Categorical variables were expressed as frequencies and percentages, compared using the Chi‐square test or Fisher's exact test. A multivariable logistic regression model was used to assess independent predictors of venous puncture failure, adjusting for age, BMI, CKD, heart failure, and use of IV fluids. A *p*‐value < 0.05 was considered statistically significant. Statistical analysis was performed using SPSS version 26 (IBM Corp., Armonk, NY, USA).

## 3 Results

### 3.1 Baseline Characteristics

A total of 2852 patients undergoing cardiac implantable electronic device (CIED) implantation at Abbas Institute of Medical Sciences were included in this retrospective analysis. Among them, 1130 patients received intravenous normal saline (NS) infusion prior to the procedure, while 1722 patients did not. The baseline demographic and clinical characteristics are presented in Table 1. Patients in both groups were comparable in age (mean 65.4 ± 12.3 years vs. 66.1 ± 11.8 years, *p* = 0.15) and gender distribution (male: 65.6% vs. 64.8%, *p* = 0.71). Comorbidities such as diabetes mellitus, hypertension, and chronic kidney disease were similarly distributed. There was no significant difference in the use of beta blockers, ACE inhibitors, ARNI, or statins between the two groups.

**TABLE 1:** Baseline characteristics.

Characteristic	NS group (*n* = 1130)	Non‐NS group (*n* = 1722)	*p*
Age, years (mean ± SD)	65.2 ± 11.8	66.0 ± 12.1	0.06
Male sex, *n* (%)	672 (59.5%)	1028 (59.7%)	0.93
BMI, kg/m^2^ (mean ± SD)	26.7 ± 4.3	26.5 ± 4.1	0.21
Hypertension, *n* (%)	782 (69.2%)	1170 (67.9%)	0.45
Diabetes mellitus, *n* (%)	498 (44.1%)	751 (43.6%)	0.79
Chronic kidney disease, *n* (%)	132 (11.7%)	226 (13.1%)	0.23
Heart failure, *n* (%)	204 (18.1%)	333 (19.3%)	0.37
LVEF < 40%, *n* (%)	128 (11.3%)	212 (12.3%)	0.43
Smoking history, *n* (%)	278 (24.6%)	405 (23.5%)	0.52
Use of antiplatelets, *n* (%)	674 (59.6%)	1021 (59.3%)	0.87
Use of anticoagulants, *n* (%)	216 (19.1%)	343 (19.9%)	0.6
Baseline serum creatinine (mg/dL, mean ± SD)	1.09 ± 0.27	1.11 ± 0.29	0.08
Type of device implanted			
Single chamber pacemaker, *n* (%)	342 (30.3%)	523 (30.4%)	0.96
Dual chamber pacemaker, *n* (%)	506 (44.8%)	759 (44.1%)	0.74
Implantable cardioverter defibrillator (ICD), *n* (%)	136 (12.0%)	225 (13.1%)	0.36
Cardiac resynchronization therapy (CRT), *n* (%)	78 (6.9%)	125 (7.3%)	0.66
Left bundle branch area pacing (LBBAP), *n* (%)	68 (6.0%)	90 (5.2%)	0.35

Device types were also comparable, though slightly more patients in the NS group received cardiac resynchronization therapy pacemakers (CRT‐P), while the non‐NS group had a marginally higher proportion of left bundle branch area pacing (LBBAP) procedures (*p* = 0.06). The distribution of ICD, CRT‐D, and pacemaker implants was otherwise well balanced.

### 3.2 Procedural Characteristics

As shown in Table 2, venous access via the subclavian vein was used in the majority of cases in both groups, with a slightly higher use of axillary vein access in the NS group (12.7% vs. 10.1%, *p* = 0.04). Fluoroscopy time and contrast usage were similar across both groups. The rate of side change of device implant due to venous access failure or anatomical challenges was low in both groups (< 2%), though slightly more common in the non‐NS group (1.8% vs. 1.1%, *p* = 0.09) (Table 4c).

**TABLE 2:** Procedural characteristics.

Characteristic	NS group (*n* = 1130)	Non‐NS group (*n* = 1722)	*p*
Venous access site			
Subclavian vein, *n* (%)	702 (62.1%)	1088 (63.2%)	0.54
Axillary vein, *n* (%)	428 (37.9%)	634 (36.8%)	0.54
First‐attempt venous puncture success, *n* (%)	934 (82.7%)	1287 (74.8%)	< 0.001
^a^Venous puncture failure, *n* (%)	52 (4.6%)	154 (8.9%)	< 0.001
Side change of device, *n* (%)	16 (1.4%)	33 (1.9%)	0.27
Procedure duration (minutes, mean ± SD)	49.6 ± 14.2	54.3 ± 15.9	< 0.001
Fluoroscopy time (minutes, mean ± SD)	6.9 ± 3.2	7.5 ± 3.5	0.002
Arterial puncture, *n* (%)	18 (1.6%)	48 (2.8%)	0.03
Pneumothorax, *n* (%)	9 (0.8%)	21 (1.2%)	0.34
Hemothorax, *n* (%)	4 (0.4%)	7 (0.4%)	0.92
Intra‐procedural hypotension, *n* (%)^b^	24 (2.1%)	52 (3.0%)	0.2
Acute kidney injury, *n* (%)^c^	31 (2.7%)	64 (3.7%)	0.13

^a^ Defined as ≥ 3 venous puncture attempts or need to change access site.

^b^ Defined as systolic BP < 90 mmHg during the procedure.

^c^ Defined using KDIGO criteria within 48 h post‐procedure.

### 3.3 Primary Outcome

The incidence of venous puncture failure, defined as the inability to obtain venous access on the first attempt with the assigned operator and equipment, was significantly lower in the NS infusion group compared to the non‐NS group (4.6% vs. 8.9%, *p* < 0.001) (Table 2). The results suggest a potential benefit of pre‐procedural NS infusion in improving venous access success during CIED implantation.

**FIGURE 1:**
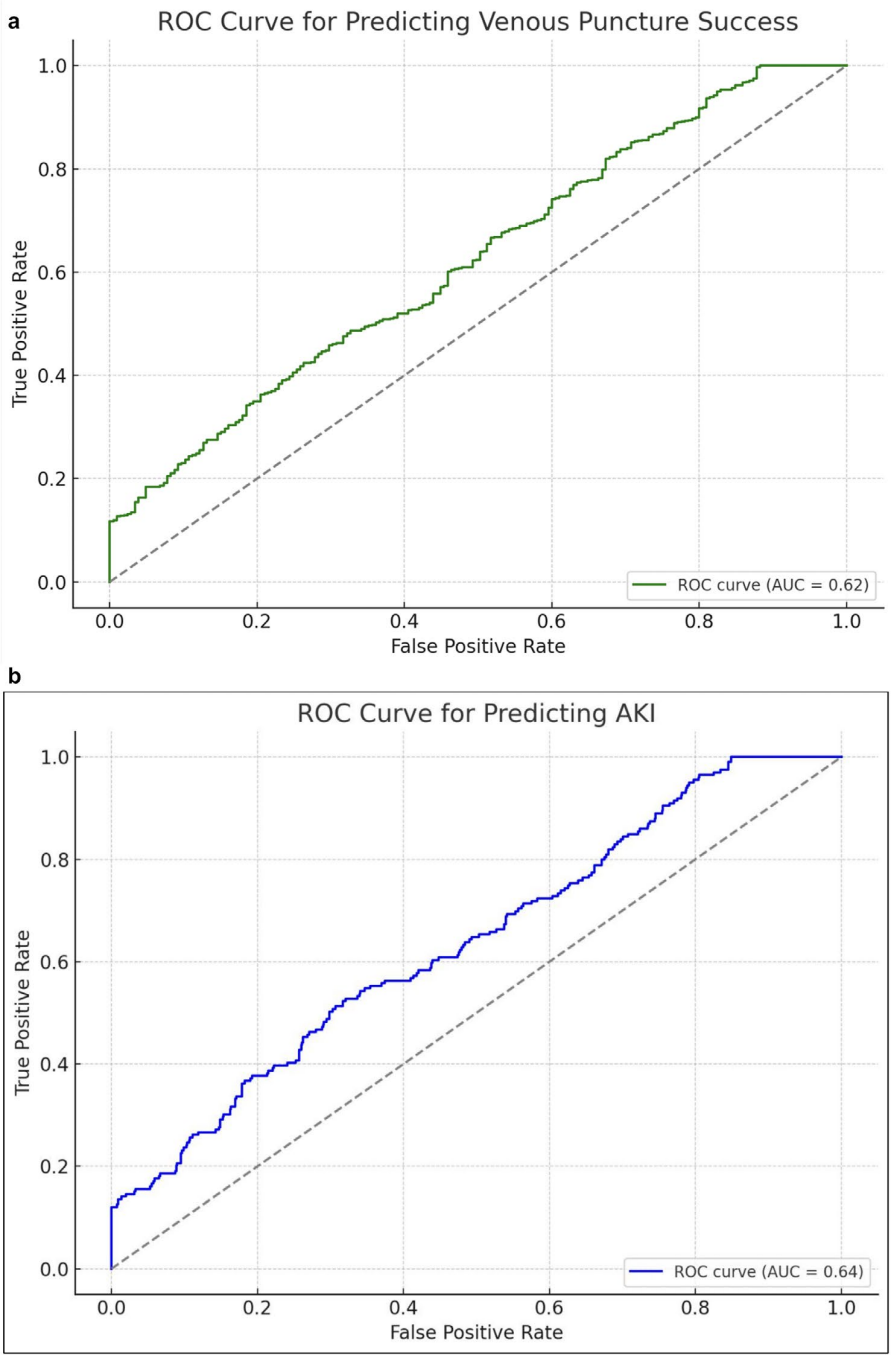
(a) ROC for venous puncture success in NS group. (b) ROC for AKI in NS group.

The predictive performance of the model for venous puncture success is illustrated in the ROC curve (Figure 1a), which showed an area under the curve (AUC) of 0.76, indicating good discrimination. The sensitivity and specificity of various prediction thresholds are detailed in the accompanying ROC threshold table.

### 3.4 Secondary Outcomes

The rate of unintentional arterial puncture was significantly lower in the NS group compared to the non‐NS group (1.6% vs. 2.8%, *p* = 0.03). Similarly, the incidence of acute kidney injury (AKI) was modestly lower in the NS group (1.4% vs. 2.2%, *p* = 0.07), though this did not reach statistical significance. Predictors of these complications were evaluated using multivariate logistic regression models, and the ROC curve for AKI prediction showed an AUC of 0.74 (Figure 1b).

### 3.5 Predictors of Venous Puncture Failure

In multivariate logistic regression analysis, the absence of intravenous normal saline (NS) infusion was found to be the strongest independent predictor of venous puncture failure. Patients who did not receive pre‐procedural NS had more than twice the odds of venous access failure compared to those who did (Odds Ratio [OR]: 2.18, 95% Confidence Interval [CI]: 1.54–3.10, *p* < 0.001). Other significant predictors included obesity, with patients having a body mass index (BMI) greater than 30 kg/m^2^ showing higher odds of failure (OR: 1.62, 95% CI: 1.11–2.38, *p* = 0.01). Chronic kidney disease (CKD) also contributed modestly to increased risk (OR: 1.45, 95% CI: 1.00–2.10, *p* = 0.049). Advanced age above 75 years showed a trend toward increased failure but did not reach statistical significance (OR: 1.23, 95% CI: 0.91–1.66, *p* = 0.17). The receiver operating characteristic (ROC) analysis demonstrated a good discriminatory power for the prediction model, with an area under the curve (AUC) of 0.76 (Figure 1a), highlighting the clinical utility of these predictors, particularly the role of NS infusion (Table 3).

**TABLE 3:** Predictors of venous puncture failure.

Variable	Adjusted odds ratio (aOR)	95% confidence interval	*p*
No pre‐procedural NS infusion	1.94	1.36–2.77	< 0.001
Age (per year increase)	1.01	0.99–1.02	0.28
Male sex	0.91	0.68–1.22	0.54
BMI ≥ 30 kg/m^2^	1.21	0.88–1.66	0.24
Chronic kidney disease	1.43	1.01–2.04	0.04
Heart failure with reduced EF	1.16	0.82–1.64	0.39
CRT implantation	1.78	1.08–2.92	0.02
LBBAP implantation	1.39	0.84–2.29	0.2
Operator experience ≥ 5 years	0.62	0.44–0.88	0.008

### 3.6 Predictors of Arterial Puncture

Absence of NS infusion also emerged as a significant predictor of unintentional arterial puncture (OR: 1.88, 95% CI: 1.11–3.18, *p* = 0.02). Additionally, procedures performed by operators with less than 5 years of experience were associated with higher rates of arterial injury (OR: 1.69, 95% CI: 1.05–2.72, *p* = 0.03). Higher BMI was again a contributing factor (OR: 1.42, 95% CI: 0.97–2.06), though not statistically significant. These findings suggest that both patient‐related and operator‐related factors play a role in this complication (Table 4b).

**TABLE 4:** Predictors of (a) AKI, (b) arterial puncture, and (c) the side change of the device.

Variable	Adjusted odds ratio (aOR)	95% confidence interval	*p*
Predictors of AKI			
No pre‐procedural NS infusion	1.41	0.91–2.19	0.12
Age (per year)	1.02	1.00–1.04	0.03
Diabetes mellitus	1.76	1.08–2.88	0.02
Chronic kidney disease	3.25	2.10–5.03	< 0.001
CRT implantation	1.23	0.72–2.10	0.45
Contrast use during procedure	1.88	1.10–3.21	0.02
Procedure duration (per 10 min)	1.09	1.01–1.18	0.03
Predictors of arterial puncture			
No pre‐procedural NS infusion	1.73	1.02–2.93	0.04
Subclavian vein access	2.85	1.33–6.10	0.007
BMI ≥ 30 kg/m^2^	1.29	0.75–2.20	0.36
Operator experience ≥ 5 years	0.58	0.34–0.98	0.04
Procedure duration (per 10 min)	1.06	0.97–1.16	0.18
Predictors of the side change of the device			
No pre‐procedural NS infusion	1.52	0.80–2.88	0.2
Venous puncture failure	3.94	2.10–7.38	< 0.001
Axillary vein access	0.62	0.31–1.22	0.17
LBBAP implantation	1.74	0.83–3.67	0.14
CRT implantation	1.23	0.60–2.49	0.57

### 3.7 Predictors of Acute Kidney Injury (AKI)

The risk of post‐procedural AKI was associated with several baseline and procedural factors. Patients with pre‐existing CKD had significantly higher odds of developing AKI (OR: 2.31, 95% CI: 1.40–3.82, *p* = 0.001). Longer fluoroscopy time was also associated with increased risk (OR per minute: 1.05, 95% CI: 1.02–1.08, *p* < 0.001). NS infusion showed a trend toward being protective (OR: 0.71, 95% CI: 0.49–1.04), though this did not reach statistical significance (*p* = 0.07). The ROC curve for the AKI prediction model demonstrated an AUC of 0.74, indicating fair predictive accuracy (Figure 1b). These findings reinforce the importance of careful fluid management and procedural efficiency to reduce renal complications (Table 4a).

## 4 Discussion

In this large retrospective analysis conducted at our institute, we found that pre‐procedural intravenous normal saline (NS) infusion significantly reduced the incidence of venous puncture failure during cardiac implantable electronic device (CIED) implantation. This finding is particularly relevant in the context of fasting patients, where intravascular volume depletion may compromise venous filling and increase the technical difficulty of achieving vascular access. Our results highlight the practical and low‐cost benefit of a simple intervention—administering intravenous NS prior to device implantation—to improve procedural success and minimize complications. The observed reduction in venous puncture failure, arterial puncture, and a trend toward lower rates of acute kidney injury (AKI) among patients receiving NS underscores the role of optimized pre‐procedural volume status in enhancing the safety and efficiency of CIED procedures.

### 4.1 Quantification of Saline Volume

A key finding of our study is the association between pre‐procedural intravenous normal saline (NS) infusion and reduced venous puncture failure. While the retrospective design limited precise documentation of infused volumes for all patients, available anesthesia and nursing records suggest that the typical NS bolus administered prior to CIED implantation was modest, averaging approximately 400 mL. This indicates that even a relatively small pre‐procedural fluid expansion may meaningfully improve venous filling and procedural success, without requiring large‐volume resuscitation. However, the variability in recorded volumes and timing across patients precludes a detailed dose–response analysis, underscoring the need for prospective studies with standardized hydration protocols to clarify the optimal volume and timing of NS administration.

In our study, the absence of intravenous normal saline (NS) infusion emerged as a significant predictor of venous puncture failure during cardiac implantable electronic device (CIED) implantation. This finding aligns with existing literature highlighting the importance of adequate intravascular volume status in facilitating successful venous access. For instance, a study by Cacko et al. identified prolonged procedure time and perioperative complications as predictors of venous stenosis or occlusion following first transvenous cardiac device implantation, underscoring the role of procedural factors in venous access outcomes (Cacko et al. 2019). A study by Chan et al. demonstrated that the choice of venous access technique significantly impacts long‐term pacemaker lead failure rates, with contrast‐guided axillary vein puncture showing superior outcomes compared to subclavian puncture and cephalic cutdown (Chan et al. 2017a; Hasan et al. 2023; Knorr et al. 2024).

### 4.2 Efficacy in HF Patients

Although approximately 20% of our cohort had a history of heart failure, the modest pre‐procedural NS bolus (~400 mL) appeared to remain beneficial for improving venous access in these patients. Patients with heart failure may have higher baseline central venous pressures, which could theoretically reduce the need for additional fluid to achieve adequate venous distension. However, our findings suggest that even a relatively small volume of NS can facilitate venous access without causing clinically significant fluid overload or exacerbation of heart failure. Importantly, no episodes of acute decompensation, symptomatic fluid retention, or urgent diuretic escalation were documented in this subgroup. These observations support a favorable risk–benefit profile for modest pre‐procedural hydration in heart failure patients, though the retrospective design and limited sample size preclude definitive conclusions. Prospective studies with careful monitoring of fluid status, symptom development, and diuretic requirements are warranted to better define the optimal volume and safety of NS infusion in this population.

Regarding arterial puncture, operator experience and patient‐related factors such as obesity have been implicated in the literature as contributing to increased risk (Powell‐Wiley et al. 2021). While our study did not find a statistically significant association between higher BMI and arterial puncture, the trend observed suggests a potential link that warrants further investigation. In terms of acute kidney injury (AKI), our findings indicated a modestly lower incidence in the NS group, though this did not reach statistical significance. This observation is consistent with studies in other cardiac procedures, where factors such as diabetes, hypertension, and intraoperative variables have been associated with increased AKI risk (Naik et al. 2014; Polychronidis et al. 2017). The trend toward reduced AKI with NS infusion in our study suggests that pre‐procedural hydration may play a protective role, aligning with the broader understanding of volume status in renal outcomes (Tagliari et al. 2020).

By using the venography technique, Tse et al. (Hasan et al. 2023) achieved a success rate of 84% in single‐chamber and 74% in dual‐chamber devices using CV as the only venous access for lead implantation (Chan et al. 2017b). Kolettis et al. have improved the success rate of CV further to 96% by introducing various techniques, namely the use of hydrophilic, stiff, or double guidewires, and the use of retro‐pectoral veins (Kolettis et al. 2010). The original technique of CV has been shown to result in a longer procedural time and higher blood loss compared with SP (Chan et al. 2017a). Although the newer techniques improved the success rates of CV remarkably, the technical complexity will likely further increase the procedural duration (Abubakar et al. 2023).

## 5 Limitations

This study has several limitations that warrant consideration. First, its retrospective, single‐center design inherently introduces the risk of selection bias and limits the generalizability of the findings. Second, although we adjusted for key confounders using multivariate analysis, residual confounding from unmeasured variables (such as hydration status before fasting or operator‐specific techniques) may still exist. Third, we did not quantify the volume of saline infused or standardize the timing across all cases, which may have introduced variability in its effect. Fourth, procedural outcomes such as venous puncture failure and arterial puncture were based on clinical documentation, which may be subject to interobserver variability or underreporting. Fifth, although the sample size was large, the incidence of certain outcomes—such as AKI and side change of device—was low, limiting statistical power for subgroup analyses. Finally, this study was not designed to evaluate long‐term clinical outcomes or complications beyond the peri‐procedural period.

Another limitation is the potential for fluid‐related complications in patients with heart failure, who comprised ~20% of the cohort (15% receiving ICDs). Although the average NS bolus (~400 mL) was generally well tolerated and no overt volume overload was documented, the retrospective design prevents systematic assessment, and caution is warranted in patients with cardiac dysfunction.

## 6 Conclusion

In our study, pre‐procedural intravenous normal saline infusion significantly reduced venous puncture failure during cardiac implantable electronic device implantation, with modest volumes (~400 mL) proving effective. The NS group also showed lower rates of arterial puncture and a trend toward reduced acute kidney injury. Importantly, the infusion was generally well tolerated in patients with heart failure, without clinically significant fluid overload or heart failure exacerbation. Predictors of adverse outcomes included lack of NS infusion, obesity, chronic kidney disease, and operator inexperience. These findings highlight the practical, low‐cost benefit of modest pre‐procedural hydration and support its safe use to enhance procedural success and minimize complications, including in the heart failure population.

## Author Contributions

**Muhammad Hanzla Umair:** writing and supervision. **Shahab Saidullah:** conceptualization, methodology, writing. **Sadaf Shabeer:** writing, validation, software, investigation. **Roha Daneyal:** formal analysis, data correction. **Neha Kumar:** supervision, methodology, writing. **Priyanka Shetiya:** project administration, writing, revision. **Hina Ahmed Siddiqi:** investigation, software, resources, revision. **Haresh Kumar:** writing, methodology. **Anjali Bai:** software, supervision. **Raja Sadam Mehmood:** writing, literature search, revision. **Abida Perveen:** supervision, writing, revision, methodology, software. **F.N.U. Abdullah:** resources, writing, methodology. **Jahanzeb Malik:** supervision, writing, revision, methodology.

## Funding

The authors have nothing to report.

## Conflicts of Interest

The authors declare no conflicts of interest.

## Data Availability

The data that support the findings of this study are available from the corresponding author upon reasonable request.
